# DNA-Protein Immunization Using *Leishmania* Peroxidoxin-1 Induces a Strong CD4^+^ T Cell Response and Partially Protects Mice from Cutaneous Leishmaniasis: Role of Fusion Murine Granulocyte-Macrophage Colony-Stimulating Factor DNA Adjuvant

**DOI:** 10.1371/journal.pntd.0003391

**Published:** 2014-12-11

**Authors:** Abebe Genetu Bayih, Nada S. Daifalla, Lashitew Gedamu

**Affiliations:** Department of Biological Sciences, University of Calgary, Calgary, Alberta, Canada; Universidade Federal de Minas Gerais, Brazil

## Abstract

**Background:**

To date, no universally effective and safe vaccine has been developed for general human use. *Leishmania donovani* Peroxidoxin-1 (LdPxn-1) is a member of the antioxidant family of proteins and is predominantly expressed in the amastigote stage of the parasite. The aim of this study was to evaluate the immunogenicity and protective efficacy of LdPxn-1 in BALB/c mice in heterologous DNA-Protein immunization regimen in the presence of fusion murine granulocyte-macrophage colony-stimulating factor (mGMCSF) DNA adjuvant.

**Methodology and Principal Findings:**

A fusion DNA of LdPxn1 and mGMCSF was cloned into a modified pcDNA vector. To confirm the expression in mammalian system, Chinese hamster ovary cells were transfected with the plasmid vector containing LdPxn1 gene. BALB/c mice were immunized twice with pcDNA-mGMCSF-LdPxn-1 or pcDNA-LdPxn1 DNA and boosted once with recombinant LdPxn-1 protein. Three weeks after the last immunization, mice were infected with *Leishmania major* promastigotes. The result showed that immunization with pcDNA-mGMCSF-LdPxn1 elicited a mixed Th-1/Th-2 immune response with significantly higher production of IFN-γ than controls. Intracellular cytokine staining of antigen-stimulated spleen cells showed that immunization with this antigen elicited significantly higher proportion of CD4^+^ T cells that express IFN-γ, TNF-α, or IL-2. The antigen also induced significantly higher proportion of multipotent CD4^+^ cells that simultaneously express the three Th-1 cytokines. Moreover, a significant reduction in the footpad swelling was seen in mice immunized with pcDNA-mGMCSF-LdPxn1 antigen. Expression study in CHO cells demonstrated that pcDNA-mGMCSF-LdPxn-1 was expressed in mammalian system.

**Conclusion:**

The result demonstrates that immunization of BALB/c mice with a plasmid expressing LdPxn1 in the presence of mGMCSF adjuvant elicits a strong specific immune response with high level induction of multipotent CD4^+^ cells that mediate protection of the mice from *Leishmania major* infection. To our knowledge, this is the first study showing the vaccine potential of *Leishmania* peroxidoxin -1.

## Author Summary

Leishmaniasis, a disease caused by protozoan parasites under the genus *Leishmania*, claims the lives of thousands of people annually. It mainly affects people in poor communities in Africa, Asia and South America. Although several drugs are available for the treatment of leishmaniasis, their efficacy is limited by the emergence of drug resistant parasite strains and by the inherent side-effects of some drugs. In light of these challenges, developing effective vaccine is considered as a crucial step in the control and ultimate elimination of the disease. In this study, we have evaluated the potential of the antioxidant *Leishmania* Peroxidoxin-1 as a candidate vaccine for leishmaniasis. The efficacy of the candidate vaccine was assessed in DNA-Protein immunization strategy in mice. We also investigated the adjuvant role of GMCSF DNA fused with the vaccine antigen in a pcDNA plasmid vector. The result showed that *Leishmania* Peroxidoxin-1 together with fusion GMCSF adjuvant in a pcDNA plasmid induces a partially protective immune response in mice. Further analysis of the immune response demonstrated that the antigen-adjuvant combination elicits CD4^+^ T cells that express IFN-γ, TNF-α, or IL-2. The antigen also induced a high frequency of CD4^+^ T cells that simultaneously express all the three cytokines. The study on samples taken from leishmaniasis patients showed that the recombinant *Leishmania* Peroxidoxin-1 protein is recognized by and elicit immune response in humans, a crucial requirement in the development of a vaccine.

## Introduction

A recent report by World Health Organization states that leishmaniasis is endemic in 98 countries in five continents. About three-fourth of these countries are developing or least developed. Thus, leishmaniasis remains to be a disease of the poor and disadvantaged [1]. *Leishmania*-HIV co-infection has become an important public health problem in places where the two infections overlap. As of 2007, 35 countries around the World have reported cases of co-infection [2], [3]. Leishmaniasis is one of the most neglected diseases and is strongly associated with poverty. Lack of access to treatment, poor housing conditions, migration of people for work or due to war and famine as well as malnutrition significantly increase morbidity and mortality due to leishmaniasis [4].

Treatment of leishmaniasis relies on chemotherapeutic agents that are mostly not effective, costly, and/or associated with serious side-effects. Although huge information is available about the immunology of leishmaniasis, no universally effective and safe vaccine has been developed against any form of leishmaniasis for general human use [5]. Different vaccine formulations have been tested against leishmaniasis including live or killed whole parasite, parasite fractions, as well as subunit vaccines in the form of DNA or recombinant proteins. The inherent inability to cause disease makes subunit vaccines the preferred forms over live or attenuated vaccines. In addition, they are relatively easy to produce and standardize. However, subunit vaccines are generally weak immunogens which necessitates repeated immunization and also the use of adjuvants. Several candidates have been extensively studied as potential *Leishmania* vaccines in the form of recombinant protein and DNA vaccines. These candidate vaccines have shown variable level of immunogenicity and protection in animal models and humans [5], [6].

Peroxidoxins (Pxns) also called peroxiredoxins or thiol-specific antioxidants are found conserved in prokaryotes and eukaryotes. By detoxifying extremely reactive oxygen intermediates such as hydroxyl radical as well as reactive nitrogen intermediates, *Leishmania* peroxidoxins play a crucial role for the parasite to evade host-defense system [7]. *Leishmania* has four peroxidoxins; Pxn1, Pxn2, Pxn3, and Pxn4. It has been shown that *Leishmania* Pxn1 and Pxn4 are predominantly expressed in the amastigote stage, whereas LPxn2 and LPxn3 are expressed more in the promastigote stage. Studies have demonstrated that peroxidoxins are highly conserved across different species of *Leishmania* [8], [9], [10].

Peroxidoxins have been used as vaccine candidates against leishmaniasis [11] and amoebiasis [12]. Peroxidoxin-2, a homolog of human thiol-specific antioxidant (TSA) from *L. major*, has been tested as a vaccine candidate against cutaneous and visceral leishmaniasis in mice and non-human primate models [11], [13], [14].

In this study, we evaluated the immunogenicity and protective efficacy of *Leishmania donovani* peroxidoxin-1 (LdPxn1) in heterologous DNA/protein immunization regimen in the presence of fusion murine Granulocyte-Macrophage Colony-Stimulating Factor (mGMCSF) adjuvant in BALB/c mice.

GMCSF is a hematopoietic growth factor that stimulates multipotent progenitor cells to differentiate into macrophages/monocytes and also to granulocytes. In addition to its role in hematopoiesis, GMCSF also functions as immunomodulator. GMCSF plays important role in activation, maturation, and function of dendritic cells. Moreover, it recruits cells such as neutrophils and monocytes to the site it is produced in a paracrine manner. As a result, GMCSF has been used as important adjuvant in infectious disease and cancer vaccine candidates [15], [16]. GMCSF has been used in the form of recombinant protein or plasmid DNA as an adjuvant in candidate vaccines for malaria [17], HIV [18], mycobacteria [19], *Leishmania* [20], as well as cancer [21]. These studies have shown that GMCSF significantly increases immunogenicity of the vaccine antigens and protection from the cognate microorganism. A GMCSF-containing therapeutic vaccine, Sipuleucel-T (Provenge, Dendreon), has been approved by US food and drug administration (FDA) for the treatment of asymptomatic or minimally symptomatic prostate cancer in men [21]. In addition to its role as a vaccine adjuvant, GMCSF can also be used as a secretory module for vaccines used in intramuscular injection. GMCSF possesses a 17 amino acid leader sequence that mediates secretion of the protein [22].

In this study, we have demonstrated that immunization of mice with LdPxn1 antigen in the presence of fusion mGMCSF adjuvant in DNA-protein prime-boost strategy induces a strong immune response with significant increase in the frequency of CD4^+^ T cells that express IFN-γ, TNF-α, and IL-2. More importantly, the antigen induced a significantly higher proportion of multipotent CD4^+^ T cells that simultaneously express all the three Th-1 cytokines and resulted in a significantly increased protection of mice from *L. major* challenge infection.

## Materials and Methods

### Mice and parasites

Four to six week old female BALB/c mice were purchased from Charles River Laboratories (Quebec, Canada). The mice were maintained under pathogen-free animal facility of the Department of Biological Sciences, University of Calgary throughout the study period. Mice were acclimatized for two weeks before immunization with the vaccine candidates. *Leishmania major* strain V1 (MHOM/IL/80/Friedlin) was obtained as a kind gift from Dr. Steven G. Reed, Infectious Disease Research Institute (IDRI) (WA, USA). In order to maintain the virulence, the parasites were obtained from the footpads of infected BALB/c mice. Stationary phase promastigotes from fresh isolates were used for the challenge experiment.

### Cells and culture media

Chinese Hamster Ovary cells (CHO) were purchased from Life Technologies (USA) and were cultured in CD CHO medium (1X) (Gibco) supplemented with HT supplement (Gibco) and L-glutamine (Gibco). M199 medium with 20% fetal bovine serum (both purchased from Gibco) was used for *L. major* culture.

### Plasmid

A modified pcDNA plasmid vector was previously developed in our lab in collaboration with Dr. Patrick Farrell, Schulich School of Engineering (Pharmaceutical Production Research Facility), University of Calgary, Canada. The plasmid DNA contains murine GMCSF (mGMCSF) and a spacer region cloned in a pcDNA 3.1 (+) frame (Invitrogen). The spacer region possesses six histidine residues, enteropeptidase cleavage site flanked by proline hinge at both ends [23], [24]. A GFP expression plasmid vector, pEGFPN3, was purchased from a commercial company (Clonetech, USA). A 20-mer CpG ODN 1826 with the sequence, 5′-tccatgacgttcctgacgtt-3′ was purchased from InvivoGen (USA).

### Cloning of *Leishmania donovani* peroxidoxin-1

LdPxn1 was cloned into modified pcDNA and pcDNA-mGMCSF plasmid vectors at *NotI* restriction site. LdPxn1 (*NotI*) F 5′- AGT CGC GGC CGC CAT GTC CTG CGG TGA CGCC-3′ and LdPxn1 (*NotI*) R 5′-AGT CGC GGC CGC TTA CTT ATT GTG ATC GACC-3′ were used as a forward and reverse primer, respectively. The primers were custom-synthesized by Alpha DNA (Quebec, Canada). PCR amplification was done as follows: one step initial DNA denaturation at 94°C for 5 minutes; thirty cycles of 1min denaturation at 94°C, 30sec annealing at 60°C, and 1min extension at 72°C; followed by 10min final extension at 72°C. The product was purified using QIAquick PCR Purification kit (QIAGEN, Canada). The PCR product and the plasmids were subjected to restriction digestion using NotI-HF (New England BioLabs, Canada). The digestion products were run in 1% low melting point agarose (Promega, Canada) and purified using QIAquick Gel Extraction kit (QIAGEN, Canada). After ligation of the PCR product and plasmids, transformation of *E. coli* DH5α was performed. The cloning was then confirmed by sequencing of plasmid DNA extracted from transformed *E. coli*.

Endotoxin-free vaccine candidate plasmid DNA was isolated from the transformed *E. coli* using EndoFree plasmid purification kit (QIAGEN, Canada) following the manufacturer's instruction. Endotoxin-free plasmid DNA samples were diluted to appropriate concentration using endotoxin-free PBS (Teknova, USA) before injection into mice.

### CHO cell transfection and western blotting

pcDNA-LdPxn1, pcDNA-mGMCSF-LdPxn1 as well as controls (pcDNA, pcDNA-mGMSCF and *pEGFPN3*) were used for transfection and expression experiment in CHO cells. Transfection of CHO cells and expression of the DNA vaccine candidates was confirmed by fluorescent microscopy and Western blotting. CHO cells were cultured in CD CHO medium supplemented with HT supplement and L-glutamine in T75CN tissue culture flasks (Sarstedt, USA) following the procedure on Gibco manual and were incubated in a 37°C incubator with 5% carbondioxide. Two sets of transfection were done; one for fluorescent microscopy and the other for Western blotting.

Cationic lipid-mediated transfection of CHO cells with DNA vaccine candidates was done using Lipofectamine 2000 transfection reagent (Invitrogen) following the manufacturer's instruction. 6×10^5^ cells were plated per well in 500 µl/well CD CHO medium in a 24-well plate (Sarstedt, USA). One to three ratio of DNA and lipofectamine were used for transfection.

Fluorescent microscopy was used to check the transfection of CHO cells after incubation for 72 hr. Cells were transfected with a mixture of each of the vaccine candidate constructs or control plasmid DNA and pEGFPN3. The expression of *GFP* by transfected CHO cells was assessed using a fluorescent microscope (Leica, DMR). Images were taken with a cooled CCDl camera (Retiga 1350 EX, Qimaging).

After checking the viability of the cells using trypan blue exclusion method (Gibco) 72 hr post-transfection, secreted and cellular proteins were isolated from the culture supernatant and cell lysate, respectively. Cells lysate was prepared by treating the cells with CHO cell lysis buffer (100 mM Tris-HCl pH 8.0, 100 mM NaCl, 10 mM EDTA, 1% Triton X-100, 2 mM PMSF) [25]. Total protein in the culture supernatant and cell lysate was precipitated using trichloroacetic acid (TCA). The protein pellets were resuspended with 1X Laemmli sample buffer (Bio-Rad, USA) with 5% fresh beta-mercaptoethanol and boiled for 5min.

Western blotting was performed on samples of culture supernatants as well as cell lysates and probed with rabbit anti-mGMCSF polyclonal antibody (AbCam, Canada) and serum from mice immunized with recombinant LdPxn1 protein. Briefly, culture supernatant (SUP) and cell lysate (Lys) samples taken from CHO cells that were transfected with pcDNA, pcDNA-mGMCSF, pcDNA-LdPxn1, pcDNA-mGMCSF-LdPxn1 and pEGFPN3 as well as rmGMCSF (AbCam, Canada) and rLdPxn1 were loaded into 12% SDS-polyacrylamide gel. After electrophoresis, the protein was transferred to a PVDF membrane using Trans-Blot Turbo transfer system (BIO-RAD, Canada). Western blotting was performed following the instruction on ECL Western blotting detection system manual (Amersham GE Healthcare, UK).

### Expression and purification of recombinant peroxidoxin-1

*Leishmania donovani* peroxidoxin 1 (LdPxn1) was previously cloned as a GST fusion and expressed in our lab by Dr. Stephen Barr [8]. For this study, Glutathione S-transferase (GST) fused recombinant peroxidoxin 1 (rLdPxn1-GST) was purified from transformed *E. coli* using Glutathione Sepharose 4B beads (Amersham GE Healthcare, UK). Endotoxin was removed from the recombinant protein using Detoxi-gel affinity Pak pre-packed columns (Pierce Biotechnology, USA) following the manufacturer's instruction. Then, the recombinant LdPxn1-GST was subjected to thrombin cleavage. About one milligram of rLdPxn1-GST in 1 ml buffer was mixed with 1X GST cleavage buffer (500 mM Tris-Cl pH 8.0, 1M NaCl, 25 mM CaCl2, and 1% beta-mercaptoethanol) and 20 U thrombin and incubated for 20 hr at room temperature. The cleaved rLdPxn1 was then purified by passing the digestion mixture through columns containing glutathione sepharose beads. Soluble Leishmania antigen (SLA) was prepared from promastigotes of L. major as described previously [26].

### Mice immunization and challenge infection

After two weeks of acclimatization, five female BALB/c mice per group were injected with DNA vaccine antigens or controls. Two DNA immunizations were given at week-0 and week-3 followed by one recombinant protein boost at week-6. All immunizations were prepared in 50 µl solution in endotoxin-free PBS (Teknova, USA). DNA immunization was performed by intramuscular injection of a mixture of 100 µg plasmid DNA and 25 µg CpG ODN in 50 µl total volume. The recombinant protein booster immunization was given to the vaccine groups by subcutaneous injection (SC) of 12.5 µg rLdPxn1 protein in combination with 25 µg CpG ODN in the right hind footpad. All the three injections to mice that received pcDNA and pcDNA-mGMCSF were given in the form of plasmid DNA only.

*Leishmania major* strain V1 (MHOM/IL/80/Friedlin) was used for the protection study in BALB/c mice. Stationary phase promastigotes, cultured in M199 medium with 20% FBS, were washed and resuspended in endotoxin-free PBS. 3×10^6^ stationary phase live promastigotes in 40 µl endotoxin-free PBS were injected subcutaneously into the hind left footpad of each mouse. The thickness of the footpads was then measured weekly until euthanasia using an electronic digital caliper (VWR, USA). Mice that showed a net footpad swelling of more than 3 mm thick or those that developed necrotic lesions were euthanized even before the end date of week-17.

### Blood collection and serum isolation

Blood samples were collected at different time points by retro-orbital sinus bleeding. At the time of euthanasia, blood was collected by cardiac puncture. Serum was isolated from whole blood samples and stored at −20°C until use.

### Spleen cell culture and stimulation

Mouse spleen was collected aseptically and cells were isolated as described previously [26]. After washing with cRPMI, cells were seeded at 2×10^5^ cells per well in 100 ul cRPMI medium in triplicate in a 96-well tissue culture plate (Sarstedt, USA). Then, the cells were stimulated with ConA (5 µg/ml), recombinant LdPxn1 protein (10 µg/ml) or *L. major* SLA (50 µg/ml). Negative control cells received medium alone. The cells were incubated for 72 hr at 37°C and 5% carbondioxide (CO_2_). After incubation, cell culture supernatant was transferred into a new plate, sealed and stored in −80°C freezer until cytokine ELISA was done.

### Measurement of antibody response

Antibody response to vaccine antigens was evaluated by measuring the magnitude of rLdPxn1-specific mouse total IgG, IgG1, and IgG2a antibody in sera from mice immunized with the vaccine antigens and controls using indirect enzyme-linked immunosorbent assay (ELISA). Moreover, the titer of IgG1 and IgG2a was determined by performing eight two-fold serial dilution of the serum.

Ninety six-well flat-bottom Nunc MaxiSorp ELISA plates (eBiosciences, USA) were coated with 5 µg/ml rPxn1 in 50 µl/well bicarbonate buffer (pH 9.6) and incubated overnight (O/N) at 4°C. Blocking was done with 200 µl/well 5% skimmed milk in 1X PBS and incubated at room temperature (RT) for 1 h. After washing 3 times with 1X PBS-Tween 20 (0.1% Tween 20 in PBS, PBST), 50 µl serum sample diluted at 1∶100 in blocking buffer was added to each well. For titration experiment, each serum sample was subjected to eight times two-fold serial dilutions. The reaction was then incubated at RT for 1 hr on a rotating Compact Rocker (Mandel, Canada). Following three washes, goat biotinylated anti-mouse IgG, IgG1 or IgG2a (SouthernBiotech, USA) secondary antibodies, Streptavidin-HRP, as well as TMB (3,3′,5,5′-tetramethylbenzidine) substrate solution were added consecutively. The reaction was stopped by adding 50 µl/well of 1N H_2_SO_4_. Finally, the absorbance was read at 450 nm using a microplate reader spectrophotometer (Molecular Devices, USA).

### Measurement of cytokine response

The level of interferon-gamma (IFN-γ) and IL-10 was measured from supernatant of antigen/mitogen stimulated and unstimulated spleen cells using BD OptEIA^ Set Mouse IFN-γ and BD OptEIA Set Mouse IL-10 (BD Biosciences, USA) kits, respectively. Cytokine ELISA was performed on the culture supernatant samples according to the manufacturer's instruction (BD Biosciences). The absorbance was read on a microplate reader (Molecular Devices, USA) and the concentration of the cytokines in the sample was calculated against the concentration of the standard using SoftMax Pro 5 software (Molecular Devices, USA).^

### Intracellular cytokine staining and flow cytometry

A seven color flow cytometry was performed on stimulated spleen cells using a three laser BD FACSAria II machine. All reagents for intracellular staining and flow cytometry were purchased from BD Biosciences (CA, USA). 1×10^6^ cells in 100 µl cRPMI per well were seeded in a flat-bottom 96-well plate (Sarstedt, USA) and stimulated with phorbol myristate acetate (PMA) (5 ng/ml)/ionomycin (500 ng/ml) (Sigma), 10 µg/ml rLdPxn1 protein antigen, 50 µg/ml *L. major* SLA or medium alone (unstimulated). Then, 2 µg/ml of antibody to CD28 was added and incubated at 37°C and 5% CO_2_ for 2 hr. Brefeldin A (GolgiPlug) was added into each well and incubated at 37°C for additional 12 hr. After blocking with 0.75 µg anti-CD16/32 (Mouse BD Fc Block) and washing, the cells were stained with V450 rat anti-mouse CD3, V500 rat anti-mouse CD4, and APC-Cy7 rat anti-mouse CD8α. The cells were then washed 2 times with staining buffer. After fixation and permeabilization with Cytofix/Cytoperm solution, the cells were washed twice with 1X BD Perm/Wash buffer. After blocking and washing, the cells were stained with PE-Cy7 rat anti-mouse IFN-γ, FITC rat anti-mouse TNF-α, PE rat anti-mouse IL-2, and APC rat Anti-mouse IL-10. Isotype control staining was done on antigen stimulated cells (in separate wells) with equal concentration of isotype-matched control of irrelevant specificity. For unstained control, antigen stimulated cells were treated with staining buffer devoid of any antibody. After washing twice, the cells were resuspended in PBS and analyzed using BD FACSAria II machine. Compensation was done using equivalent mixture of BD CompBeads Anti-Rat Ig, κ and BD CompBeads Negative Control (FBS) following the manufacturer's instruction. The result was analyzed using FlowJo software (Tree Star, Inc, USA). The lymphocytes were gated based on the size, granularity, as well as surface and intracellular staining profiles.

### Immunogenicity experiment on human samples

To determine the immunogenicity of rLdPxn1 in humans, venous blood was collected from cutaneous leishmaniasis (CL) and visceral leishmaniasis (VL) patients in Ethiopia. In addition to leishmaniasis patients, healthy controls were also included in the study. Specimens from a total of eight CL and ten VL patients as well as nine healthy (uninfected) controls were used to assess the immunogenicity of the antigens in humans. Ten to fifteen millilitres of venous blood was collected from each individual using heparinized vacutainer tubes. The plasma was isolated and stored in -20°C freezer until use for antibody ELISA. Peripheral blood mononuclear cells (PBMCs) were isolated from the blood samples using Ficoll-Paque Plus (GE Healthcare) gradient centrifugation. The cells were then washed three times with RPMI 1640 medium and resuspended in 20% FBS in RPMI 1640. After counting, the cells were mixed with freezing medium (RPMI 1640 20% FBS 10% DMSO (Sigma) and stored in −80°C freezer.

Total human IgG specific rLdPxn1 was measured from plasma samples following the ELISA procedure described above. The level of cytokine response was assessed by measuring IFN-γ and IL-10 from supernatants of stimulated PBMCs using sandwich ELISA. PBMCs were stimulated with rLdPxn1 protein, *L. major* SLA, or phytohaemagglutinin (PHA). A negative control well contained PBMCs in the absence of antigen/mitogen.

### Ethical clearance

The experimental protocol for the mice study (BI 2006-33) was reviewed and approved by the Life and Environmental Sciences Animal Care Committee (LESACC), The University of Calgary. The experiments were done in accordance with the principles by The Canadian Council on Animal Care. The procedure for human work was approved by The Conjoint Health Research Ethics Board, The University of Calgary and The National Health Research Ethics Review Committee (NERC), The Federal Democratic Republic of Ethiopia. Blood samples were collected after obtaining written informed consent from the study participants.

### Statistical analysis

The statistical differences between different groups of mice were analyzed using Kruskal-Wallis test. Whereas, the difference between means of any two groups was compared using Mann-Whitney U test. A *p*-value of less than 0.05 was considered statistically significant. All statistical analysis was done using IBM SPSS Statistics 20 software.

## Results

### The DNA vaccine antigens are expressed in mammalian cells and GMCSF facilitates their secretion

In order to confirm expression of the vaccine antigens in mammalian cells, CHO cells were transfected with the DNA vaccine constructs and expression of the genes was assessed by Western blotting. Co-transfection of CHO cells with plasmid DNA containing the vaccine antigens and pEGFPN3 confirmed that the cells were successfully transfected with the antigens. Cells that expressed GFP appeared green on a fluorescent microscope (S1 Figure).

Western blotting on culture supernatant and lysate of cells transfected with LdPxn1 plasmid DNA confirmed that the vaccine antigens are expressed in mammalian system. Moreover, GMCSF facilitates the secretion of the vaccine antigen. S2A Figure shows the expression of pcDNA-LdPxn1 in CHO cells. Probing with anti-LdPxn1 serum produced signals at the expected LdPxn1 size of 22kDa on both culture supernatant (SUP) and lysate (LYS) samples taken from pcDNA-LdPxn1 transfected CHO cells (lanes 3 and 4, respectively). A more intense band appeared on the lysate sample (lane-4) than that of culture supernatant (lane-3). The recombinant LdPxn1 protein control gave a positive signal of slightly lower size (lane-8). However, no band was seen on samples taken from pcDNA-mGMCSF-Pxn1 transfected cells (S2A Figure, lane-5 and -6).

S2B Figure shows the expression of pcDNA-mGMCSF-Pxn1 in CHO cells (lane-5 and-6). Upon probing with anti-mGMCSF antibody, the sample taken from culture supernatant gave a protein band of about 52kDa (lane-5) while the lysate sample produced a slightly smaller band (lane-6). pcDNA-mGMCSF control was also expressed with a size of 25 to 30kDa and most of the protein was secreted into the culture supernatant (lane-1). As expected, no band was seen on samples from pcDNA-LdPxn1 transfected cells (lane-3 and -4) (S2B Figure).

### *Leishmania* peroxidoxin-1 induces specific antibody response in BALB/c mice

**Figure 1:**
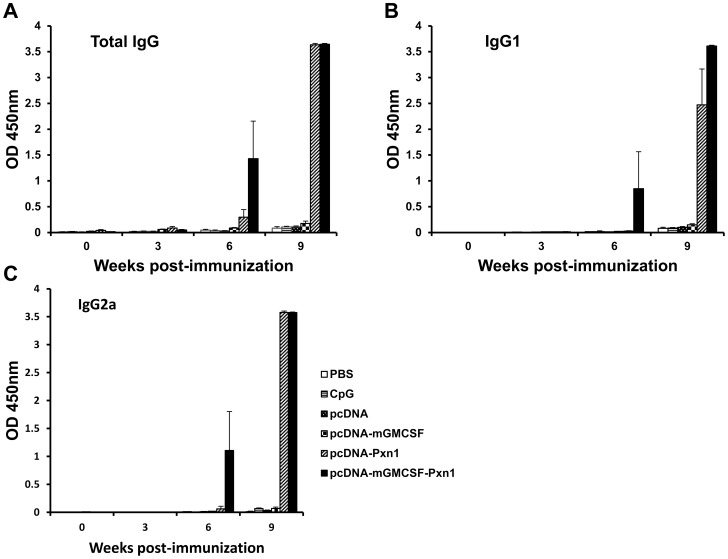
*Leishmania* Peroxidoxin 1-specific antibody response in BALB/c mice before challenge. Mice were immunized twice with DNA antigens and controls in the presence of CpG ODN adjuvant followed by a boost with the recombinant LdPxn1. All immunizations were given in three week intervals. Blood samples were collected before immunization, at the time of each immunization and upon euthanasia. Total IgG (A), IgG1 (B), and IgG2a (C) were measured using ELISA and the result is depicted as mean OD_450nm_ of five mice per group and standard error of the mean (SEM). Statistical comparison between groups was performed using Mann-Whitney U test. The assay was done in duplicate wells for each mouse serum. This is one of two experiments with similar result.

Antigen-specific antibody response was measured from sera collected at different time points after immunization of mice with the plasmid expressing LdPxn1 antigen followed by recombinant protein boost. At Week-6 post-immunization, mice that were immunized with plasmids expressing peroxidoxin-1 DNA in the presence or absence of mGMCSF fusion induced significantly higher antigen-specific total IgG response than the respective controls, pcDNA or pcDNA-mGMCSF (p<0.05). Although not statistically significant, the presence of mGMCSF fusion appreciably increased the total IgG response as compared to immunization with the antigen in the absence of GMCSF. Three weeks after recombinant protein boost (Week-9), a dramatic increase in antibody response was seen in mice primed with the DNA vaccine antigens in the presence or absence of fusion GMCSF (Fig. 1A).

Like total IgG response, mice primed with plasmids expressing the vaccine antigens developed a significantly higher IgG1 response than the control mice that received pcDNA or pcDNA-mGMCSF (p<0.05) (Fig. 1B). On the other hand, only mice that were immunized with the plasmid expressing LdPxn1 in the presence of mGMCSF fusion showed significantly higher IgG2a response at Week-6 post immunization (p<0.05). Immunization with pcDNA-LdPxn1 devoid of mGMCSF fusion did not induce significantly higher IgG2a response than the respective control at this time point. However, at week-9, the mice that were immunized with pcDNA-LdPxn1 or pcDNA-mGMCSF-LdPxn1 showed significantly higher IgG2a response than the respective controls (p<0.05) (Fig. 1C).

**Figure 2:**
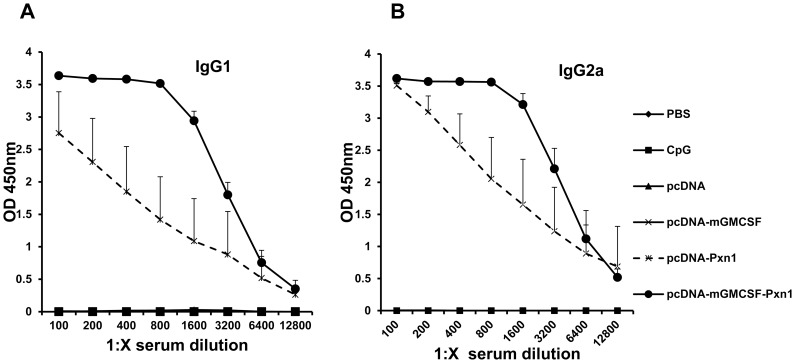
Titration of *Leishmania* Peroxidoxin 1-specific antibody response in BALB/c mice before challenge. Blood samples were collected at Week-9 after the first immunization. The level of IgG1 (A) and IgG2a (B) was measured using ELISA in a two-fold serially diluted sera. The result is depicted as mean OD_450nm_ of five mice per group. Statistical comparison between groups was performed using Mann-Whitney U test. The assay was done in duplicate wells for each mouse serum. This is one of two experiments with similar result.

Titration of sera from immunized and control mice showed that immunization with the plasmid expressing peroxidoxin-1 antigen in the presence of fusion mGMCSF induced higher antibody response than the antigen without mGMCSF (Fig. 2).

### GMCSF fusion increases antigen-specific cell-mediated immune response in BALB/c mice

Cell-mediated immune response to the vaccine antigens was assessed by measuring the level of IFN-γ and IL-10 produced by antigen-stimulated spleen cells isolated from mice that were immunized with the vaccine antigens or controls.

**Figure 3:**
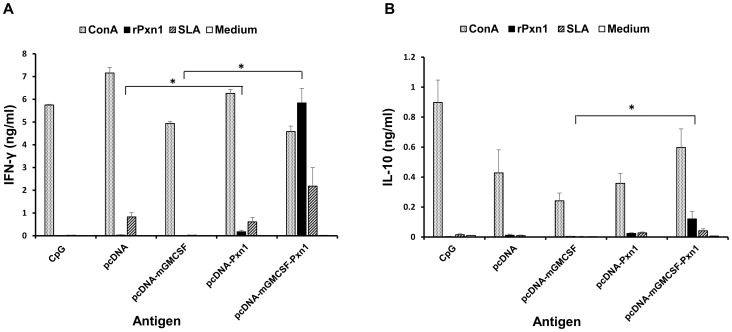
Pre-challenge cytokine response of mice immunized with peroxidoxin-1 antigen in DNA/protein prime-boost immunization regimen. IFN-γ (A) and IL-10 (B) production in stimulated spleen cells was measured using cytokine ELISA. Mice immunized twice with pcDNA-LdPxn1 or pcDNA-mGMCSF-LdPxn1 were boosted with rLdPxn1 in the presence of CpG ODN. The control mice received three doses of pcDNA, pcDNA-mGMCSF, or CpG ODN alone. Spleen cells were isolated from mice (five mice per group) three weeks after the last immunization and stimulated *in vitro* with concanavalin A (5 µg/ml), recombinant LdPxn1 (Ag) (10 µg/ml), or soluble *Leishmania* antigen (SLA) (50 µg/ml). Unstimulated cells were included as negative control. Cells were cultured at 37°C and 5% CO_2_ and culture supernatant was collected at 72 hr. The level of IFN-γ and IL-10 was measured using cytokine ELISA kit (BD Biosciences). The concentration of the cytokines (ng/ml) was calculated by correlating the optical density to concentration of the protein standard included in the kit. The mean concentration and standard error of the mean (SEM) of five mice per group is shown. Statistical comparison between groups was performed using Mann-Whitney U test. Asterisks indicate statistically significant difference in the cytokine production between mice immunized with antigen and the respective control (p<0.05). The assay was done in duplicate wells for each mouse serum. This is one of two experiments with similar result.

As shown in Fig. 3, immunization with two doses of LdPxn1 expressing plasmid DNA in the presence or absence of fusion mGMCSF followed by a recombinant LdPxn1 protein boost induced significantly higher IFN-γ response than immunization with the controls, pcDNA and pcDNA-mGMCSF (P<0.05). In addition to IFN-γ, spleen cells isolated from mice immunized with pcDNA-mGMCSF-LdPxn1 produced significantly more IL-10 than those from control mice (P<0.05). The IFN-γ to IL-10 ratio was 7.2 and 29.6 for the mice immunized with pcDNA-LdPxn1 and pcDNA-mGMCSF-Pxn1, respectively. On the other hand, injection of pcDNA and pcDNA-mGMCSF controls resulted in a ratio of only 2.2 and 2.7, respectively.

### LdPxn1 antigen induces CD4^+^ T cells that express IFN-γ, TNF-α, and IL-2

In order to dissect the cytokine profile of antigen-stimulated spleen cells from immunized mice, the phenotype of cytokine producing T cells was assessed using a multiparametric flow cytometry. Antigen-stimulated lymphocytes were gated into different subsets based on their surface and intracellular cytokine staining profiles (S3 Figure). The magnitude and quality of cytokine response of these cells was expressed in terms of the percentage of cytokine expressing T cells and median fluorescent intensity (MFI) of each cytokine. The product of the frequency of these cells and MFI, also known as integrated MFI (iMFI), was calculated to evaluate the overall quality of the immune response in mice that were immunized with the antigens in comparison with those that received the controls.

**Figure 4:**
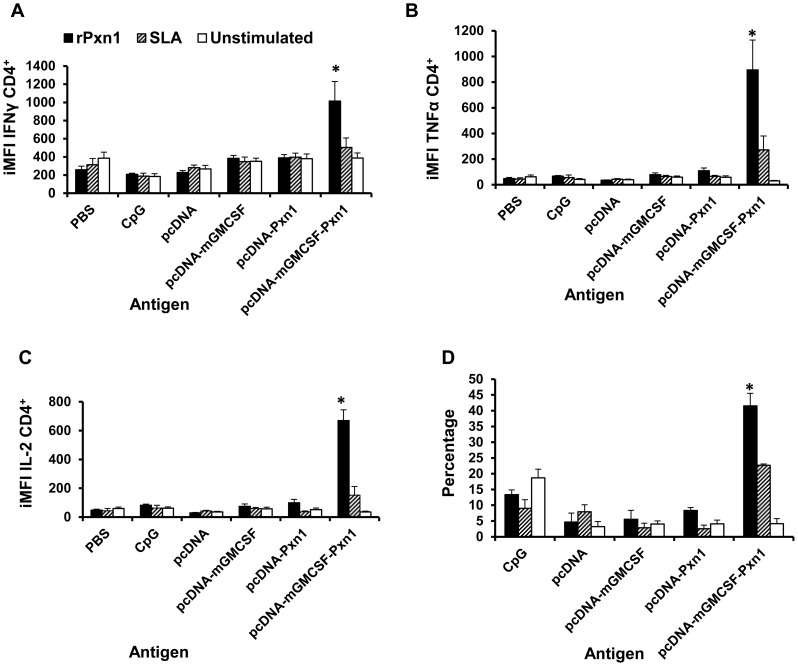
Antigen-specific cytokine expressing CD4^+^ T cells at the time of challenge. Integrated median fluorescent intensity (iMFI) of IFN-γ (A), TNF-α (B), and IL-2 (C) as well as the percentage of multipotent CD4^+^ T cells that simultaneously express IFN-γ ^+^, TNF-α ^+^, and IL-2 ^+^ (D). Integrated MFI was calculated as a product of the percentage of the cytokine producing CD4^+^ T cells and the median fluorescent intensity of the cytokine. Mice immunized twice with pcDNA-LdPxn1 or pcDNA-mGMCSF-LdPxn1 were boosted with rLdPxn1 in the presence of CpG ODN. The control mice received three doses of pcDNA, pcDNA-mGMCSF, or CpG ODN alone. Asterisks indicate statistically significant difference between cells from antigen immunized mice and controls (p<0.05).

The result clearly shows that immunization with two doses of pcDNA-mGMCSF-LdPxn1 DNA followed by a single booster immunization with rLdPxn1 protein induced significantly higher CD4^+^ T cell response with high level expression of IFN-γ, TNF-α, and IL-2 (Fig. 4). However, no appreciable difference was seen between the immunized and control mice with respect to cytokine production by antigen-stimulated CD8^+^ T cells.

With regard to IFN-γ expressing CD4^+^ T cells, immunization with pcDNA-LdPxn1 induced significantly higher response than the control, pcDNA (p<0.05). However, no significant difference was seen between immunization with pcDNA-mGMCSF-LdPxn1 and the control pcDNA-mGMCSF in the frequency of IFN-γ producing CD4^+^ T cells. A different picture was seen with regard to the MFI values. That is, only immunization with the antigen in the presence of mGMCSF induced a significantly higher MFI than the control (p<0.05). Thus, as shown in Fig. 4A, immunization with the plasmid expressing LdPxn1 in the presence of mGMCSF resulted in the highest iMFI for IFN-γ, which is significantly higher compared to the controls and also the plasmid expressing the antigen without mGMCSF fusion (p<0.05).

Analysis of TNF-α expression in antigen stimulated CD4^+^ T cells shows that immunization with the plasmid expressing LdPxn1 antigen in the presence or absence of fusion mGMCSF adjuvant induced significantly higher frequency of TNF-α ^+^ CD4^+^ T cells than controls (p<0.05). However, significantly higher degree of expression of this cytokine was seen only in CD4^+^ T cells from mice that were immunized with the plasmid expressing the antigen in the presence of mGMCSF adjuvant. As a result, the highest iMFI for TNF-α was seen in mice that were immunized with pcDNA-mGMCSF-LdPxn1. The difference was statistically significant as compared to immunization with pcDNA-LdPxn1 alone or with control preparations (p<0.05) (Fig. 4B)

Like TNF-α, the IL-2 response of CD4^+^ T cells is highest in cells that were isolated from mice immunized with pcDNA-mGMCSF-LdPxn1. The response is more pronounced in terms of MFI and iMFI (Fig. 4C). With regard to the frequency of IL-2 expressing CD4^+^ T cells, the plasmid expressing LdPxn1 with or without mGMCSF induced significantly higher response compared to the controls (p<0.05).

Unlike Th-1 cytokine response, no clear difference was seen between immunized mice and controls with regard to IL-10 expression.

In order to analyse the proportion of multipotent T cells that simultaneously express more than one cytokine, we gated IFN-γ expressing CD4^+^ T cells further into TNF-α and IL-2 expressing subsets (S3G Figure). Analysis of these cells shows that there is no significant difference between antigen immunized mice and controls with regard to the proportion of CD4^+^ T cells that simultaneously expressing IFN-γ and TNF-α or IFN-γ and IL-2. Whereas, the data clearly demonstrate that cells from mice immunized with pcDNA-mGMCSF-LdPxn1 had significantly higher proportion of CD4^+^ T cells that simultaneously express all the three Th-1 cytokines compared to the controls (p<0.05). The response in these mice was also significantly higher than that of mice immunized with plasmid expressing the antigen in the absence of mGMCSF (p<0.05) (Fig. 4D).

### A strong specific antibody- and cell-mediated immune response is maintained after *L. major* infection

In order to assess the durability of LdPxn-1-specific immune response after *L. major* infection, we measured the level of specific antibody in the serum and cytokine in the stimulated spleen cells. We also assessed the magnitude of cell-mediated response by measuring the cytokine expressing T cells using flow cytometry.

**Figure 5:**
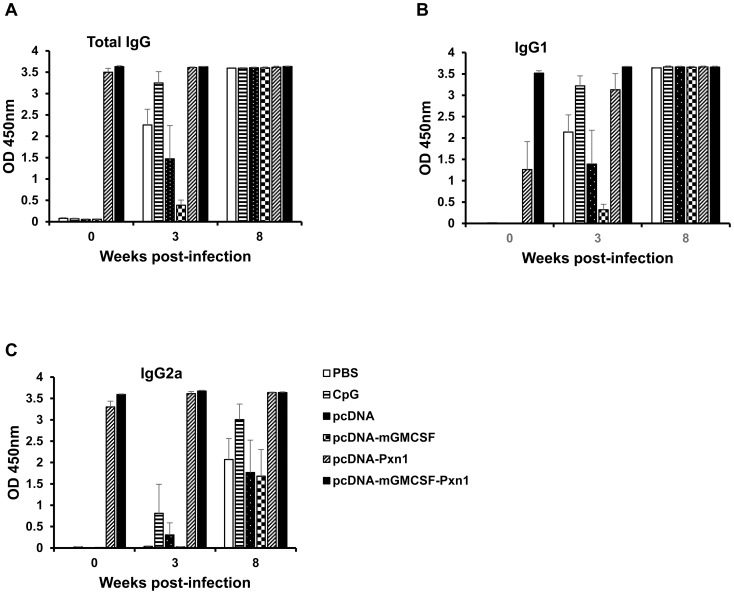
*Leishmania* Peroxidoxin 1-specific antibody response in BALB/c mice after infection with *L. major*. Peroxidoxin 1-specific total IgG (A), IgG1 (B), and IgG2a (C) antibody response after infection with *L. major*. The antibody response was measured using ELISA and the result is depicted as mean OD_450nm_ of five mice per group and standard error of the mean (SEM). The assay was done in duplicate wells for each mouse serum.

As shown in Fig. 5, the LdPxn1-specific antibody response remained high after infection. Mice that received the plasmids expressing LdPxn1 antigen showed high IgG, IgG1 and IgG2a response before infection as well as three and eight weeks after infection. These mice produced a remarkably higher IgG2a response than control mice (Fig. 5C). The LdPxn1-specific antibody response in the control groups appears to be predominantly IgG1.

**Figure 6:**
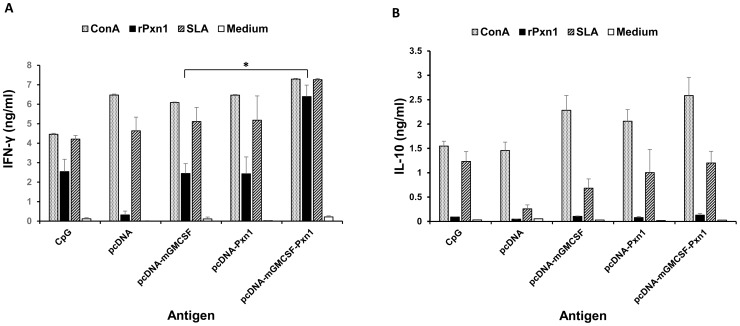
Cytokine response of mice immunized with peroxidoxin-1 antigen in DNA/protein prime-boost immunization regimen eight weeks post-infection. IFN-γ (A) and IL-10 (B) production in stimulated spleen cells was measured using cytokine ELISA. Mice immunized twice with pcDNA-LdPxn1 or pcDNA-mGMCSF-LdPxn1 were boosted with rLdPxn1 in the presence of CpG ODN. The control mice received three doses of pcDNA, pcDNA-mGMCSF, or CpG ODN alone. Mice were infected with stationary phase *L major* three weeks after the last immunization. Cytokine ELISA was done on antigen-stimulated and control spleen cells isolated from mice at eight weeks after infection. The data represent the mean cytokine concentration of five mice per group. Statistical comparison between groups was performed using Mann-Whitney U test. Asterisks indicate statistically significant difference in the cytokine production between mice immunized with antigen and the respective control (p<0.05).

Like antibody response, cell-mediated response remained high eight weeks after *L. major* infection (Fig. 6). Antigen-stimulated spleen cells from mice immunized with pcDNA-mGMCSF-LdPx1 produced significantly higher IFN-γ than the control mice (Fig. 6A). Unlike, pre-challenge response, no difference was seen between immunized and control mice regarding IL-10 production (Fig. 6B).

**Figure 7:**
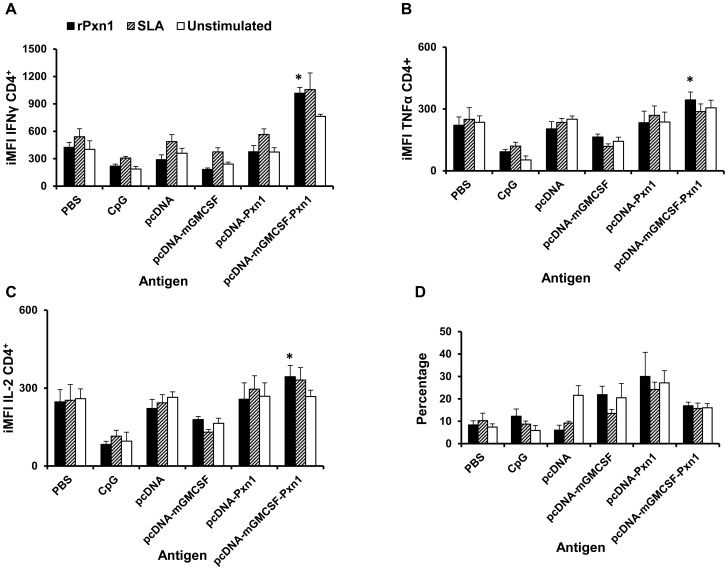
Antigen-specific cytokine expressing CD4^+^ T cells eight weeks post-infection. Integrated median fluorescent intensity (iMFI) of IFN-γ (A), TNF-α (B), and IL-2 (C) as well as the percentage of multipotent CD4^+^ T cells that simultaneously express IFN-γ ^+^, TNF-α ^+^, and IL-2 ^+^ (D). Mice immunized twice with pcDNA-LdPxn1 or pcDNA-mGMCSF-LdPxn1 were boosted with rLdPxn1 in the presence of CpG ODN. The control mice received three doses of either pcDNA, pcDNA-mGMCSF, or CpG ODN alone. Spleen cells from immunized and control mice were isolated eight weeks post-infection and stimulated with rLdPxn1 or SLA. Asterisks indicate statistically significant difference between cells from antigen immunized mice and controls (p<0.05).

Multiparametric flow cytometer analysis of stimulated spleen cells shows that mice immunized with pcDNA-mGMCSF-LdPx1 kept high IFN-γ, TNF-α, and IL-2 response eight weeks post-infection (Fig.7). The response was significantly higher than that of controls. However, the proportion of multipotent CD4^+^ cells in immunized mice is not different from that of controls (Fig. 7C).

### Peroxidoxin-1 antigen results partial protection of mice from *Leishmania major* infection

**Figure 8:**
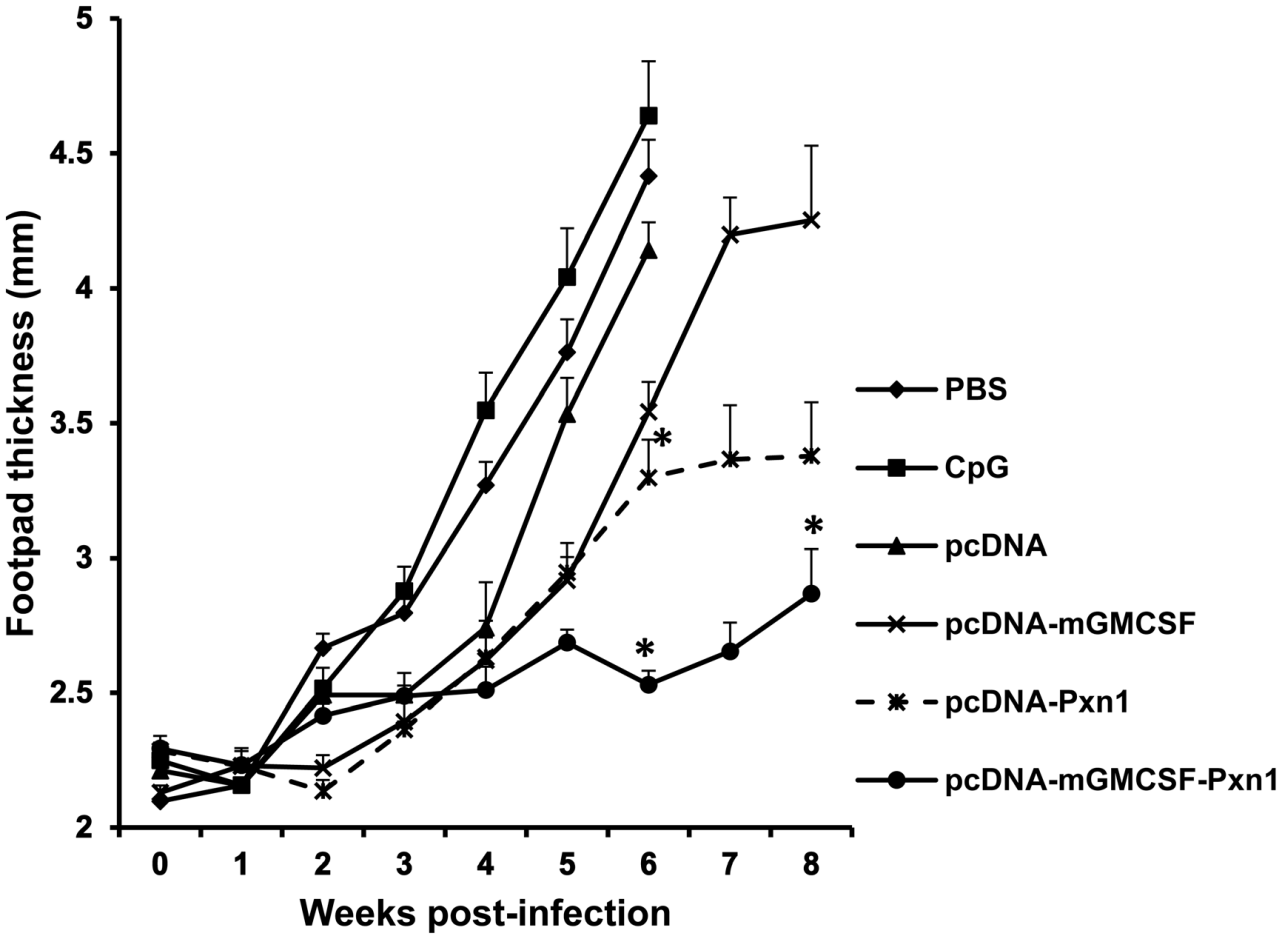
Footpad swelling of mice immunized with antigens and controls in DNA/protein immunization strategy and infected with *L. major*. BALB/c mice (five per group) were immunized twice with DNA antigens and controls in the presence of CpG ODN adjuvant followed by a boost with recombinant LdPxn1 protein. All immunizations were given in three week intervals. At Week-9, all mice were infected on the footpad with subcutaneous injection of 3 × 10^6^ stationary phase *L. major* promastigotes in the hind footpad. The footpad swelling was assessed by measuring the thickness of infected footpad weekly for eight weeks using electronic digital caliper. The data represents mean footpad size in millimetre of five mice and standard error of the mean. Asterisks indicate statistically significant difference in footpad swelling between antigen immunized mice and controls (p<0.05).

In order to evaluate the protective efficacy of the vaccine antigens, footpad swelling was measured every week for eight weeks after challenge infection with *Leishmania major*. As shown in Fig. 8, control mice that received pcDNA vector alone, pcDNA-mGMCSF, CpG alone, or saline (PBS) developed the highest footpad lesion that progressively increased over time. Most of the control mice had to be euthanized at Week-6 post-challenge because they developed lesions with a net increase of about 3mm in thickness. On the other hand, mice that were immunized with vaccine antigens generally developed lower footpad lesion over the entire period of the experiment. Mice primed with the plasmid expressing LdPxn1 in the presence of mGMCSF fusion DNA adjuvant followed by a booster immunization with rLdPxn1 showed the least footpad swelling (Fig.8).

At Week-6 post challenge, mice immunized with plasmid expressing the antigen with or without mGMCSF adjuvant developed significantly smaller lesion than that of the respective control groups (p<0.05). Likewise, at Week-8 post infection, mice that were immunized with pcDNA-mGMCSF-Pxn1 had significantly smaller lesions than those that were immunized with the control DNA, pcDNA-mGMCSF (p<0.05) (Fig. 8).

### *Leishmania* peroxidoxin-1 is immunogenic in humans

To be considered as a vaccine candidate, an antigen should be recognized by and elicit immune response in the host that the vaccine antigen is intended to be used. Induction of immune response in experimental animals such as mice does not always guarantee the effectiveness of the antigen in humans [5], [27]. Therefore, we tested the immunogenicity of rLdPxn1 protein using sera collected from cutaneous (CL) and visceral leishmaniasis (VL) patients and PBMCs of CL patients.

**Figure 9:**
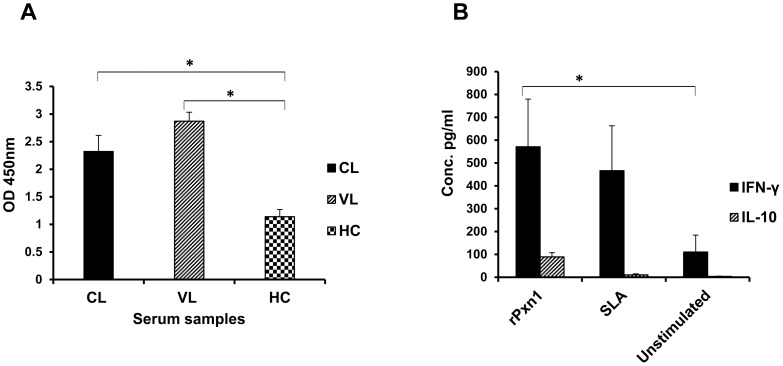
Immunogenicity of rLdPxn1 in cutaneous and visceral leishmaniasis patient samples. (A) The level of LdPxn1-specific total IgG measured from plasma collected from cutaneous (n = 8) and visceral leishmaniasis patients (n = 10) as well as healthy individuals (n = 9) using antibody ELISA. The result was expressed as mean OD_450nm_ and standard error of the mean. Statistical comparison between groups was performed using Mann-Whitney U test. “HC” refers to “Healthy Controls”. (B) The level of IFN-γ and IL-10 in rLdPxn1-stimulated peripheral blood mononuclear cells isolated from venous blood of cutaneous leishmaniasis patients (n = 8). Positive and negative control cells were included by stimulation with phytohaemagglutinin (PHA) or adding medium only, respectively. The result represented as mean concentration of IFN-γ and IL-10 and standard error of the mean. Statistical comparison between groups was performed using Mann-Whitney U test. Stimulation with PHA gave 1510.4±67.3 pg/ml IFN-γ and 380.3±77.7 pg/ml IL-10. Asterisks indicate statistically significant difference between: CL/VL patient samples and healthy controls (A) or rLdPxn1 stimulated and unstimulated PBMCs from CL patients (B) (p<0.05). The antibody ELISA assay was done in triplicate wells for each patient's serum sample.

Fig. 9A shows the level of antigen-specific IgG response in the sera of cutaneous and visceral leishmaniasis patients as well as those of healthy controls. The level of human IgG specific to rLdPxn1 in both cutaneous and visceral leishmaniasis patient samples was significantly higher than that of healthy controls (p<0.05).

Cytokine ELISA in PBMCs of CL patients showed that rLdPxn1 is immunogenic in humans. *In vitro* stimulation of PBMCs with rLdPxn1 protein resulted in significantly higher IFN-γ and IL-10 than unstimulated cells (p<0.05). Moreover, stimulation with *L major* SLA also showed high level IFN-γ response (Fig. 9B).

## Discussion

In this study, we have found that *Leishmania* Peroxidoxin-1 DNA fused with mGMCSF elicits strong immune response and partially protects mice from *Leishmania major* infection when administered in DNA-protein prime-boost immunization strategy. The antigen induces specific CD4^+^ T cells that express Th-1 cytokines such as IFN-γ and TNF-α. Moreover, the preliminary data showed that the recombinant *Leishmania* peroxidoxin-1 protein is also recognized by the human immune system as exhibited by high degree of antigen-specific antibody in the sera of leishmaniasis patients as compared to healthy individuals.

Unlike *Leishmania* peroxidoxin 2 (also called TSA), peroxidoxin 1 detoxifies not only toxic oxygen radicals but also nitrogen intermediates. Thus, it plays an important role in the survival of the parasite inside macrophages. Furthermore, peroxidoxin 1 is predominantly expressed in the amastigote stage of the parasite. Hence, we proposed that LdPxn1 could be a good vaccine candidate against leishmaniasis. It has been shown in other studies that parasite proteins that are expressed predominantly in the amastigote stage of the parasite are generally more useful antigens than their counterparts that are exclusively or predominantly expressed in the promastigote stage. In human host, promastigotes exist only in the early stage of infection. Amastigotes, on the other hand, persisted throughout the infection period and are the main target of the host immune system [27], [28], [29]. Moreover, dendritic cells (major antigen presenting cells) take up amastigotes but not promastigotes [30].

In this study, we investigated the immunogenicity and protective efficacy of LdPxn1 in DNA/protein immunization strategy in BALB/c mice. We also examined the adjuvant role of GMCSF in the form of fusion DNA with the vaccine antigen. We proposed that fusing GMCSF with the vaccine antigen has dual advantages. First, it acts as a secretory module to secrete the vaccine antigen from transfected muscle cells and makes the recombinant protein antigen amenable to processing and presentation by professional antigen presenting cells. Secondly, GMCSF itself acts as an adjuvant. GMCSF is a chemoattractant factor and immunomodulator. As a chemoattractant factor, it recruits neutrophils and monocytes to the site where it is produced. As immunomodulator cytokine, it activates dendritic cells and mediates their maturation. Thus, we hypothesized that GMCSF could play an important role in enhancing the efficacy of the vaccine antigen. GMCSF has been used as an adjuvant in vaccine candidates against various infectious diseases [15], [16], [17] as well as in a cancer candidate vaccine [21].

In this study, we used heterologous prime-boost immunization. This strategy is more effective than homologous one in inducing stronger immune response and better protection. Several studies have clearly shown that heterologous prime boost immunization is more effective than homologous one [31], [32]. Mazumder and colleagues [32] clearly demonstrated that DNA/protein heterologous immunization of *L. donovani* gp63 induced increased Th-1 type immune response than the homologous DNA/DNA or protein/protein immunization strategies as shown by increased production of IFN-γ and IL-12, with concomitant increase in nitric oxide production. This immunization strategy also showed significantly higher degree of protection against *L. major* infection.

In general, the magnitude and quality of immune response induced by *Leishmania* vaccine antigen(s) before infection is considered to determine the efficacy of the antigen(s) in protecting against infection with the parasite. In order to assess the type and degree of pre-challenge immune response, we conducted an immunogenicity study by measuring antigen-specific antibody and cell-mediated responses prior to infecting the mice with *L. major* promastigotes. Multiparameter flow cytometry was also performed to further analyze the quality of cell-mediated immune response that the vaccine antigens induced.

DNA/protein immunization with LdPxn1 has shown that the antigen is immunogenic in mice. Moreover, based on the proportion of pre-challenge IgG2a and IgG1, the study shows that the vaccine antigens induced a mixed Th1/Th2 response when given in DNA/protein strategy in BALB/c mice.

The higher antibody response before recombinant boost in mice that were immunized with pcDNA-mGMCSF-LdPxn1 than those that received the antigen without mGMCSF indicates that the presence of mGMCSF fusion increases the immune response to the antigens. This could be due to the secretory role and/or adjuvancy of mGMCSF. A study on the effect of secretion of proteins on antibody response showed that a secreted protein produced 18-times more antibodies than non-secreted form of the same protein [33].

Titration experiment using pre-infection sera showed that immunization with plasmid expressing peroxidoxin 1 in the presence of mGMCSF showed higher titers of both IgG1 and IgG2a than the antigen in the absence of mGMCSF. Other studies have also demonstrated similar results where protective antigens induced high titer of both IgG1 and IgG2a [14], [34], [35].

A more direct way of evaluating the protective immune response of a *Leishmania* vaccine antigen is measuring the degree and quality of cell-mediated immune response before challenge infection. Susceptibility and resistance to *Leishmania* is mediated by different cytokines. Th-1 cytokines such as IFN-γ, TNF-α, and IL-2 favor resistance to infection while IL-4, IL-10 and IL-13 mediate disease progression and parasite persistence. Interferon gamma and TNF-α work synergistically to induce the leishmanicidal activity of macrophages through the production of nitric oxide. On the other hand, IL-10 inhibits IFN-γ-mediated activation of macrophages [5], [36], [37]. Generally, production of high level Th-1 cytokines at the time of infection predicts protection against *L. major* infection. Recent studies have demonstrated that the degree of induction of multifunctional CD4^+^ T cells that simultaneously produce multipleTh-1 cytokines is a better correlate of protection against experimental *Leishmania major* infection in mice [38], [39]. Therefore, in our study, we assessed the efficacy of the vaccine antigens by measuring the amount of IFN-γ and IL-10 using ELISA and also by evaluating the expression of IFN-γ, TNF-α, IL-2, and IL-10 cytokines on antigen-stimulated spleen cells using multiparameter flow cytometry.

Cytokine production before infection showed that the plasmid expressing LdPxn1 antigen in the presence or absence of mGMCSF fusion induced the production of both IFN-γ and IL-10. Previous studies demonstrated that the presence of high level of IFN-γ at the time of infection predicts the protective effect of the antigen against infection by *L. major* [34]. Other studies demonstrated that the protective efficacy of vaccine antigens not only depends on the amount of IFN-γ but also IL-10 at the time of challenge infection [40]. Thus, high IFN-γ to IL-10 ratio at the time of challenge is a better correlate of protection. The results of this study agree with these findings. At the time of infection, mice immunized with the more protective pcDNA-mGMCSF-LdPxn1 antigen had a IFN-γ to IL-10 ratio of 4- and 11-fold higher than mice immunized with the less protective pcDNA-LdPxn1 and non-protective controls, respectively.

This study also shows that the high level expression of three Th-1 cytokines namely IFN-γ, TNF-α and IL-2 by antigen-stimulated spleen cells at the time of infection correlates with the level of protection against *L. major* infection in mice. This was elucidated by the results of multiparameter flow cytometry in CD4^+^ T cells. Helper T cell type-1 (Th-1) cytokine expression on CD4^+^ T cells prior to *L. major* infection clearly demonstrates that immunization with pcDNA-mGMCSF-Pxn1 induced the highest expression of each of the Th-1 cytokines, IFN-γ, TNF-α and IL-2 (Fig. 4). Taken together, immunization with the plasmid expressing LdPxn1 in the presence of mGMCSF resulted in high quality Th-1 cytokine response. This correlates with the highest protective efficacy of the antigen.

Recent studies have shown that the degree of induction of multifunctional Th-1 cells is a better predictor of protection against *L. major* infection in mice. These cells simultaneously express IFN-γ, TNF-α, and IL-2 [38], [39]. In our study, we found that immunization with pcDNA-mGMCSF-Pxn1 induced the highest frequency of IFN-γ^+^TNF-α^+^IL-2^+^ CD4^+^ T cells and showed the highest level of protection of mice from *L. major* challenge infection.

Unlike CD4^+^ response, the cytokine response of CD8^+^ T cells before infection did not demonstrate clear reflection of the protection data. That is, the vaccine antigens that brought protection did not show as significantly higher response as seen in CD4^+^ T cells. Taken together, based on flow cytometry data on pre-challenge samples, the increase protection conferred by pcDNA-mGMCSF-LdPxn1 antigen appears to be mediated by CD4^+^ T cells that express IFN-γ, TNF-α and IL-2 individually or simultaneously.

Assessment of the antigen-specific antibody and cytokine response after *L. major* challenge infection also strengthened the result of the pre-challenge experiment. That is, immunization with pcDNA-mGMCSF-LdPxn1 followed by a boost with rLdPxn1 resulted in a durable response that remained high eight weeks post-infection. After *L. major* infection, the antigen-specific antibody response remained high in mice that were immunized with the vaccine antigen. Mice that received some of the control preparations also produced high level LdPxn1-specific total IgG and IgG1. However, the amount of IgG2a in these mice is appreciably lower than that of antigen immunized ones. Hence, mice immunized with the vaccine antigens showed much higher IgG2a to IgG1 ratio than the control mice at three- and eight-weeks post-infection indicating the maintenance of higher Th-1 response in antigen immunized mice than controls. Moreover, the IFN-γ response in the mice that were immunized with pcDNA-mGMCSF-LdPxn1 remained high eight-weeks post-infection. However, the IL-10 response in these mice waned after infection and was not different from control mice. Likewise, the proportion of CD4^+^ cells that individually express IFN-γ, TNF-α and IL-2 remained high eight-weeks post infection. These findings demonstrate that the LdPxn1-specific response remains high several weeks after challenge infection with *L. major*.

Taken together, antibody and cytokine responses of mice that were immunized with the vaccine antigens indicate that the mGMCSF-containing antigen is more immunogenic than the same antigen without mGMSCF. Moreover, mGMCSF containing LdPxn1 showed higher frequency of CD4^+^ T cells expressing Th-1 cytokines than the antigen without mGMCSF. It is, therefore, safe to conclude that the presence of mGMCSF fusion increased immunogenicity of the vaccine antigens.

Based on our data, we do not exactly know what feature of mGMCSF enabled it to potentiate the immunogenicity and protection of the antigen. Both the secretory and immunomodulatory roles of mGMCSF are important features that could enable the molecule to potentiate the vaccine antigens. Farrell and co-workers [24] demonstrated that the increased production of antibody by GMCSF fusion is the result of secretory role rather than activity (adjuvancy). In our study, we confirmed the secretory role of mGMCSF when fused with a vaccine antigen. However, we did not directly investigate the immunomodulatory effect of the molecule.

In this study, we administered CpG ODN together with the vaccine antigens. It has been shown in several studies that bacterial CpG ODN enhances the immune response to the vaccine antigens. Moreover, it skews the immune response to a Th-1 type and favors induction of a protective cellular immune response against intracellular infections such as *Leishmania* [32], [41], [42].

Confirmation of the expression of the antigens in mammalian cells is a prerequisite for the use of any DNA vaccine candidate. To do this, we transfected CHO cells with the vaccine antigens and investigated the expression of the antigens by Western blotting. *Leishmania donovani* peroxidoxin 1 gene was cloned in a modified pcDNA plasmid under CMV promoter. The CMV promoter allows efficient expression on the vaccine antigens. The plasmid vector also contains mGMCSF gene upstream of the multiple cloning site.

We proposed that, in addition to being a cytokine adjuvant, GMCSF can also mediate the secretion of the fused vaccine antigen proteins. It has been shown in other studies that GMCSF is effectively secreted out from mammalian cells [22]. Intramuscular (IM) injection of plasmid DNA primarily transfects myocytes. As these cells lack the antigen presentation machinery, the antigen has to reach to professional antigen presenting cells for an effective immune response to be initiated [43]. It has been shown in other studies that GMCSF is effectively secreted out from mammalian cells [23].

Our transfection experiment showed that the LdPxn1 DNA antigen is expressed in CHO cells. Moreover, we confirmed that GMCSF mediates the secretion of the vaccine antigen. Western blotting experiment on cells that were transfected with plasmids containing mGMCSF resulted in recombinant proteins larger in size than predicted by their amino acid content. We believe that the difference between the predicted and actual sizes is attributed to the size increase due to glycosylation of mGMCSF. Similar results were seen in other studies involving expression of GMCSF in eukaryotic expression systems [22], [44], [45], [46], [47].

It is not clear why probing with anti-LdPxn1 anti-sera did not produce any signal on samples from pcDNA-mGMCSF-Pxn1 transfected cells (S2A Figure). This could be due to masking of specific epitopes of LdPxn1 by mGMCSF. Other studies showed a similar result [48].

Granulocyte-macrophage colony-stimulating factor (GMCSF) protein possesses a 17 amino acid leader sequence that mediates secretion of the protein [22]. In our study, we have shown that fusing the vaccine antigen with mGMCSF enhances secretion of the vaccine antigen from transfected cells. As shown in S2B Figure, mGMCSF-fused LdPxn1 is predominantly secreted into the culture medium.

In order to be considered as a vaccine, potential candidate antigens should be recognized by and elicit immune response in the human host. Induction of response in experimental animals such as mice does not always guarantee effectiveness in humans [5], [27]. Moreover, different vaccine antigens induce variable levels of recognition by human host. For example, it has been demonstrated that recombinant cysteine protease B (rCPB) is better recognized by sera from visceral leishmaniasis patients than rCPA [49]. Therefore, we assessed the immunogenicity of rLdPxn1 in Ethiopian cutaneous (CL) and visceral leishmaniasis (VL) patient samples.

The result of the preliminary experiment on leishmaniasis patients and healthy controls shows that LdPxn1 protein is recognized by the human immune system. Moreover, it shows that the antigen cross-reacts with peroxidoxins of other *Leishmania* species such as *L. aethiopica*, the predominant cause of cutaneous leishmaniasis in Ethiopia. Cytokine response against visceral leishmaniasis patient PBMCs was not investigated due to logistical problem. The VL samples were collected in a remote field site where there was no tissue culture or cell preservation facility.

In conclusion, our study has shown that *Leishmania* peroxidoxin-1 in the presence of fusion murine GMCSF DNA adjuvant induces partial protection against cutaneous leishmaniasis in mice. The level of protection was strongly correlated with induction of a high level expression of IFN-γ, TNF-α and IL-2 cytokines by antigen-specific CD4^+^ T cells. Induction of high level specific immune response and of pre-challenge multipotent CD4^+^ T cells, partial protection of genetically susceptible BALB/c mice from high dose infection with *L. major*, recognition by CL and VL patient samples as well as its predominant expression in amastigote stage of the parasite make Peroxidoxin-1 one of the promising anti-leishmanial vaccine candidates.

## Supporting Information

S1 Figure **Fluorescent microscopy of CHO cells co-transfected with antigen construct and pEGFPN3.** (A) Bright Field (B) Green Fluorescent Field.
(TIF)

S2 Figure **Western blotting of samples from CHO cells transfected with LdPxn1 gene cloned in pcDNA and pcDNA-mGMCSF.** Cell culture supernatant (SUP) and cell lysate (LYS) proteins of transfected CHO cells were run on 12% denaturing polyacrylamide gel and Western blotting was done using: (A) Pooled sera from mice immunized with pcDNA-Pxn1 and ECL-anti-mouse IgG-peroxidase primary and secondary antibodies, respectively, and (B) Rabbit-anti-mGMCSF and ECL-anti-rabbit IgG-HRP (donkey) primary and secondary antibodies, respectively and Lanes: 1) pcDNA-mGMCSF-SUP, 2) pcDNA-mGMCSF-LYS, 3) pcDNA-Pxn1-SUP, 4) pcDNA-Pxn1-LYS, 5) pcDNA-mGMCSF-Pxn1-SUP, 6) pcDNA-mGMCSF-Pxn1-LYS, 7) pEGFPN3-SUP, 8) Recombinant LdPxn1 protein.
(TIF)

S3 Figure **Gating strategy used in multiparameteric flow cytometry.** Spleen cells were isolated from immunized mice and controls (five mice per group) and stimulated *in vitro* with recombinant LdPxn1 (Ag) (10 µg/ml), *Leishmania major* soluble *Leishmania* antigen (SLA) (50 µg/ml) or phorbol myristate acetate (PMA) (5 ng/ml)/ionomycin (500 ng/ml). Unstimulated cells were included as negative control. Cells were cultured at 37°C and 5% CO_2_. Stimulated and unstimulated cells were stained first with a cocktail of V450 rat anti-mouse CD3, V500 rat anti-mouse CD4, and APC-Cy7 rat anti-mouse CD8α followed by another cocktail containing PE-Cy7 rat anti-mouse IFN-γ, FITC rat anti-mouse TNF-α, PE rat anti-mouse IL-2, and APC rat Anti-mouse IL-10. Multicolor flow cytometry was then performed using FACSAria II machine (BD, USA). Data was analysed using FlowJo software (Tree Star Inc, USA). Gating of different lymphocyte populations was performed based on surface marker and intracellular cytokine expression. CD3^+^ cells were gated from the total lymphocyte population (A and B). In turn, CD4^+^ and CD8^+^ cells were gated from CD3^+^ cell population (C). Then, the frequencies of CD4^+^ IFN-γ ^+^ (D), CD4^+^ TNF-α ^+^ (E), CD4^+^ IL-2 ^+^ (F) were determined. The proportion of multifunctional Th-1 cells was determined by subdividing IFN-γ expressing cells further into TNF-α and IL-2 expressing cells (G). The total IFN-γ, TNF-α, IL-2 cytokine producing cells in the spleen for this sample is 5.6×10^4^, 6.0×10^4^, and 6.9×10^4^, respectively.
(TIF)
