# Supplementary figures and images for: DNA-Protein Immunization Using Leishmania Peroxidoxin-1 Induces a Strong CD4+ T Cell Response and Partially Protects Mice from Cutaneous Leishmaniasis: Role of Fusion Murine Granulocyte-Macrophage Colony-Stimulating Factor DNA Adjuvant
Source: PLoS Negl Trop Dis. 2014 Dec 11;8(12):e3391. doi: 10.1371/journal.pntd.0003391 (PMC4263403; doi:10.1371/journal.pntd.0003391)

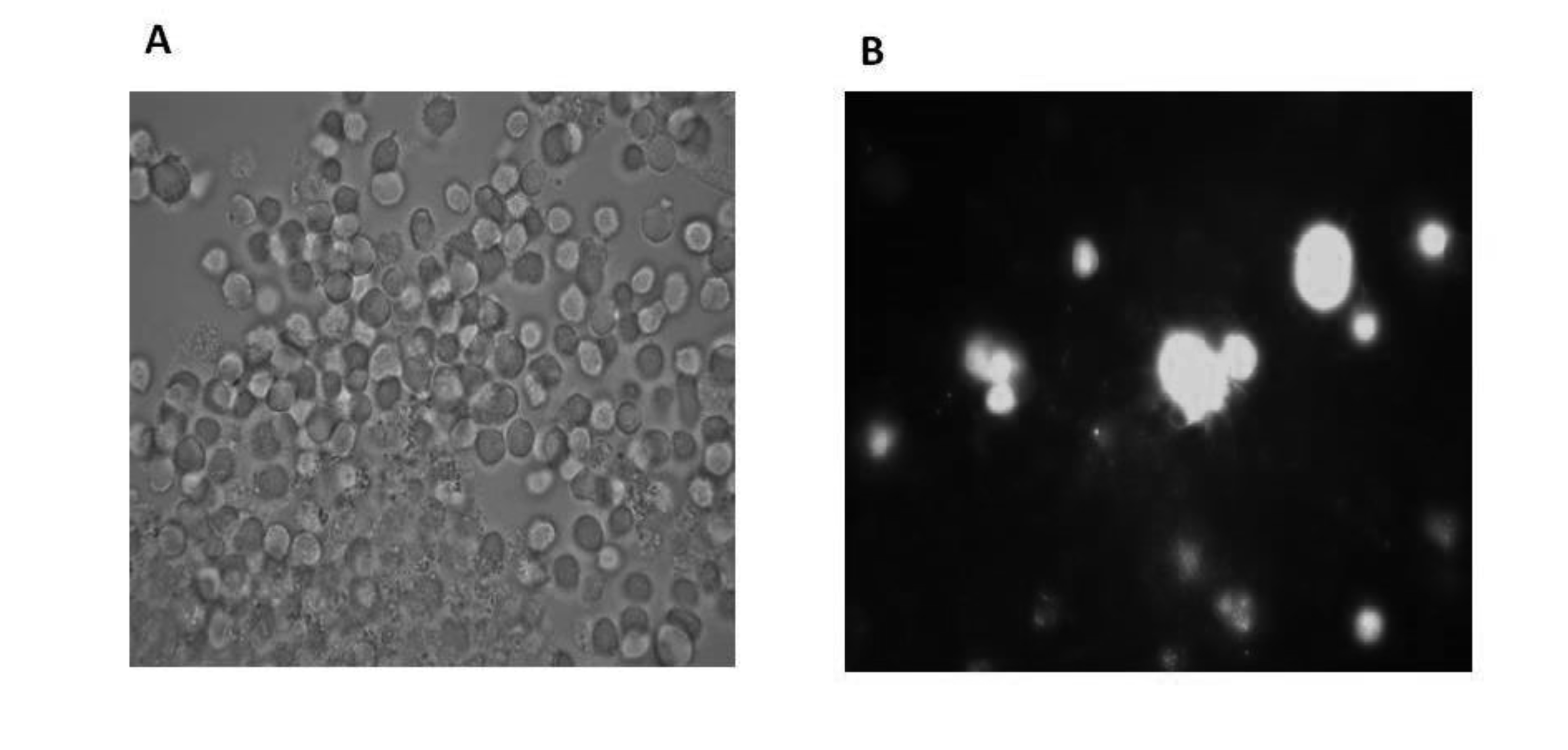

Supplement: S1 Figure — Fluorescent microscopy of CHO cells co-transfected with antigen construct and pEGFPN3. (A) Bright Field (B) Green Fluorescent Field. (TIF) [file pntd.0003391.s001.tif]

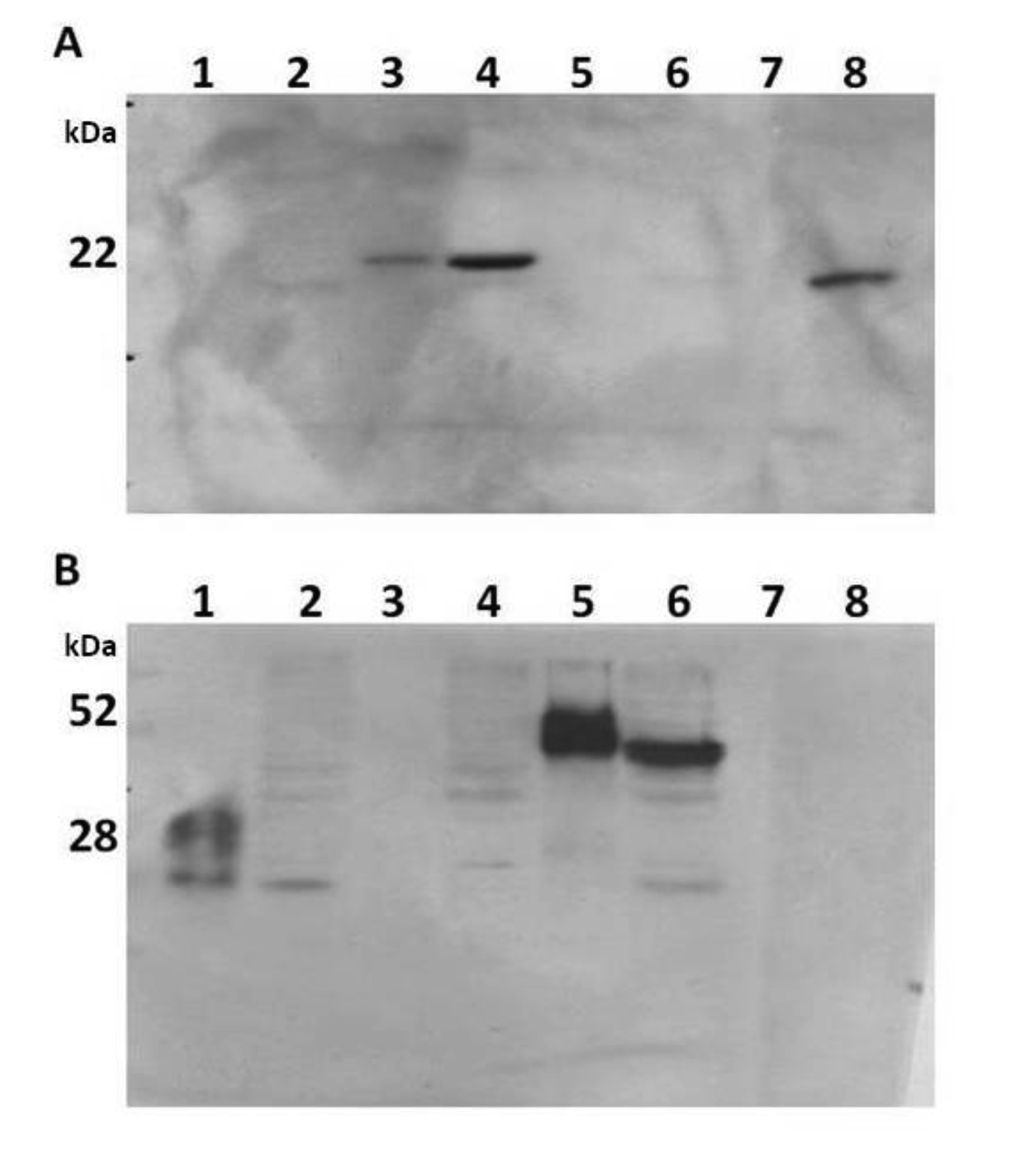

Supplement: S2 Figure — Western blotting of samples from CHO cells transfected with LdPxn1 gene cloned in pcDNA and pcDNA-mGMCSF. Cell culture supernatant (SUP) and cell lysate (LYS) proteins of transfected CHO cells were run on 12% denaturing polyacrylamide gel and Western blotting was done using: (A) Pooled sera from mice immunized with pcDNA-Pxn1 and ECL-anti-mouse IgG-peroxidase primary and secondary antibodies, respectively, and (B) Rabbit-anti-mGMCSF and ECL-anti-rabbit IgG-HRP (donkey) primary and secondary antibodies, respectively and Lanes: 1) pcDNA-mGMCSF-SUP, 2) pcDNA-mGMCSF-LYS, 3) pcDNA-Pxn1-SUP, 4) pcDNA-Pxn1-LYS, 5) pcDNA-mGMCSF-Pxn1-SUP, 6) pcDNA-mGMCSF-Pxn1-LYS, 7) pEGFPN3-SUP, 8) Recombinant LdPxn1 protein. (TIF) [file pntd.0003391.s002.tif]

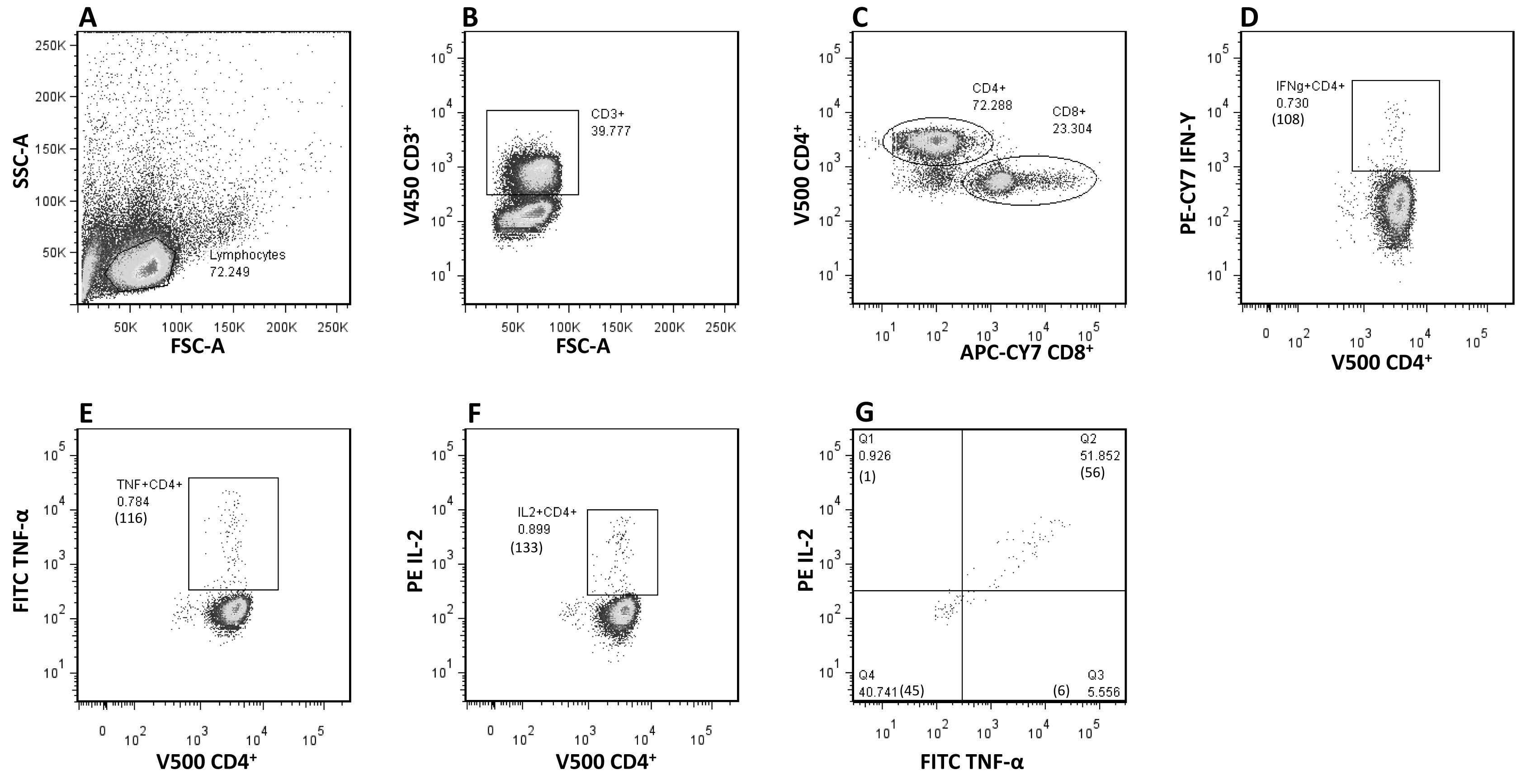

Supplement: S3 Figure — Gating strategy used in multiparameteric flow cytometry. Spleen cells were isolated from immunized mice and controls (five mice per group) and stimulated in vitro with recombinant LdPxn1 (Ag) (10 µg/ml), Leishmania major soluble Leishmania antigen (SLA) (50 µg/ml) or phorbol myristate acetate (PMA) (5 ng/ml)/ionomycin (500 ng/ml). Unstimulated cells were included as negative control. Cells were cultured at 37°C and 5% CO2. Stimulated and unstimulated cells were stained first with a cocktail of V450 rat anti-mouse CD3, V500 rat anti-mouse CD4, and APC-Cy7 rat anti-mouse CD8α followed by another cocktail containing PE-Cy7 rat anti-mouse IFN-γ, FITC rat anti-mouse TNF-α, PE rat anti-mouse IL-2, and APC rat Anti-mouse IL-10. Multicolor flow cytometry was then performed using FACSAria II machine (BD, USA). Data was analysed using FlowJo software (Tree Star Inc, USA). Gating of different lymphocyte populations was performed based on surface marker and intracellular cytokine expression. CD3+ cells were gated from the total lymphocyte population (A and B). In turn, CD4+ and CD8+ cells were gated from CD3+ cell population (C). Then, the frequencies of CD4+ IFN-γ + (D), CD4+ TNF-α + (E), CD4+ IL-2 + (F) were determined. The proportion of multifunctional Th-1 cells was determined by subdividing IFN-γ expressing cells further into TNF-α and IL-2 expressing cells (G). The total IFN-γ, TNF-α, IL-2 cytokine producing cells in the spleen for this sample is 5.6×10^4^, 6.0×10^4^, and 6.9×10^4^, respectively. (TIF) [file pntd.0003391.s003.tif]
